# Where and when are portion sizes larger in young children? An analysis of eating occasion size among 1·5–5-year-olds in the UK National Diet and Nutrition Survey (2008–2017)

**DOI:** 10.1017/S1368980021005024

**Published:** 2022-12

**Authors:** Alice Porter, Zoi Toumpakari, Ruth Kipping, Carolyn Summerbell, Laura Johnson

**Affiliations:** 1 Population Health Sciences, Bristol Medical School, University of Bristol, Bristol, UK; 2 Centre for Exercise, Nutrition and Health Sciences, School for Policy Studies, University of Bristol, Bristol BS8 2BN, UK; 3 Department of Sport and Exercise Sciences, Durham University, Durham, UK; 4 Fuse, NIHR Centre for Translational Research in Public Health, London, UK

**Keywords:** Portion size, Eating occasions, Young children, National Diet and Nutrition Survey

## Abstract

**Objective:**

To identify eating occasion-level and individual-level factors associated with the consumption of larger portions in young children and estimate their relative importance.

**Design:**

Cross-sectional.

**Setting:**

Data from parent-reported 4-d food diaries in the UK National Diet and Nutrition Survey (2008–2017) were analysed. Multilevel models explored variation in eating occasion size (kJ) within (*n* 48 419 occasions) and between children (*n* 1962) for all eating occasions. Eating contexts: location, eating companion, watching TV, and sitting at a table and individual characteristics: age, gender, ethnicity and parental socio-economic status were explored as potential correlates of eating occasion size.

**Participants:**

Children aged 1·5–5 years.

**Results:**

Median eating occasion size was 657 kJ (IQR 356, 1117). Eating occasion size variation was primarily attributed (90 %) to differences between eating occasions. Most (73 %) eating occasions were consumed at home. In adjusted models, eating occasions in eateries were 377 kJ larger than at home. Eating occasions sitting at a table, *v*. not, were 197 kJ larger. Eating in childcare, with additional family members and friends, and whilst watching TV were other eating contexts associated with slightly larger eating occasion sizes.

**Conclusions:**

Eating contexts that vary from one eating occasion to another are more important than demographic characteristics that vary between children in explaining variation in consumed portion sizes in young children. Strategies to promote consumption of age-appropriate portion sizes in young children should be developed, especially in the home environment, in eating contexts such as sitting at the table, eating with others and watching TV.

Childhood obesity is a worldwide public health problem, with 38 million children under the age of 5 years classified as overweight or obese in 2019^(1)^. Large portion sizes are suggested to contribute to childhood obesity^(2)^.

Experimental evidence has established a link between serving large portions and greater energy intake (EI) in young children, defined as the ‘portion size effect’^(3,4)^. The effect has been observed for meals and snacks, and across consecutive days^(5)^, however may vary depending on the individual, food or environment^(6,7)^. To better understand this variability, we need to explore which factors are associated with the consumption of large portions in children. Several factors such as genetic susceptibility, responsiveness to food, parent feeding styles and the home food environment have been proposed to increase a child’s behavioural susceptibility to consuming large portions^(7)^ and increase weight^(8,9)^. Existing research has focused on individual factors and less is known about within-person factors such as eating environments.

Observational studies add to the experimental literature by exploring portion sizes in free-living settings and in larger, more diverse samples. The National Diet and Nutrition Survey (NDNS) is a nationally representative cross-sectional survey, which collects dietary data from children and adults in the UK^(10)^. Although data on served portions are not collected, the data provide estimates of portions consumed. These data can be used to explore potential factors associated with the intake of larger portions, a more proximal factor on the proposed causal pathway from larger servings to excessive consumption and subsequent weight gain.

Previous studies using NDNS data have observed associations with the consumption of individual foods, in children and adolescents^(11–14)^. Consuming larger portions (g) of energy-dense foods such as chocolate, confectionary, savoury snacks and biscuits was associated with eating out of the home and watching TV, being older, male and having lower household income. Eating out of home and with friends was also associated with greater non-core EI (kcal) (e.g soft drinks, savoury snacks and chocolate) in adolescents^(13)^. Larger consumed portions of vegetables (g) were observed during the weekend and the evening meal, whilst eating at home and among older children^(12)^. Not watching TV and sitting at a table were also associated with greater vegetable consumption (g)^(14)^. These studies provide insight into which eating contexts and individual characteristics may lead to the consumption of larger portion of individual foods. Consuming larger portions of low energy-dense foods, such as fruit and vegetables, can be beneficial for children’s health^(15)^. In a meal, increasing the portion of fruit or vegetables will increase the volume (g) of the portion but, owing to their low energy density, may decrease the total energy consumed (kJ/kcal) from that meal^(16)^. Overall meal size (kcal) (regardless of food type) has been prospectively associated with excessive weight gain in young children^(17)^. Therefore, it is important to explore associations in relation to the overall energy content of eating occasions (referred to hereafter as eating occasion size), where foods and beverages are consumed in combination.

We also need to understand the relative importance of eating environments *v*. individual characteristics. In previous studies, 89 % of variability in non-core food intake^(13)^ and 82 % of variability in consumed vegetable portions^(12)^ were attributed to differences between eating occasions. This suggests targeting high-risk environments could be more effective if prioritised over specific person-level characteristics. Understanding whether variability in eating occasion size is attributable to differences between eating occasions or between young children, and the eating contexts and individual characteristics associated with larger eating occasions could help us to understand when, where and for who the risk of consuming larger portions is higher.

Young children eat in distinct environments, with typically less control over their food choices than older children, and may have increased susceptibility to the portion size effect^(18)^. Although many portion size guidance resources aimed at feeding young children (referred to as 1 to 5 years of age) are available in the UK, many are not informed by the portion size research^(19)^. Therefore, exploring factors associated with portion size could help contribute to the call for improvement of nutrition guidelines that are research-driven, contextually specific and based on causal mechanisms^(20)^.

This study aimed to describe young children’s eating occasions and to explore the relative contributions of *within*-children (between eating occasions) and *between*-children variation in eating occasion size. We aimed to identify possible eating contexts and individual characteristics associated with larger eating occasion size (kJ) in young children (aged 1 to 5 years).

## Methods

### Study sample

Secondary data analysis was conducted on dietary data from 1962 young children aged 1·5–5 years in the UK NDNS Years 1 to 9 (2008/2009–2017) rolling programme. The survey design has been described elsewhere^(21)^. NDNS data were downloaded from the UK Data Archive^(22)^.

### Dietary data

Dietary data were collected via 4-d estimated food diaries, completed by parents of the participating children. Parents were asked to record all foods and beverages consumed, including the day and exact time. Parents estimated portion sizes using household measures (e.g. tablespoons), grams from packaging and example pictures provided^(23)^. Diaries were coded by a trained NDNS research team. Where grams were not reported, portion sizes were determined by coders using household measures in the Diet In Nutrients Out system^(24)^ or available packaging. Portion sizes were converted into energy by the NDNS research team using the food composition data from the Department of Health NDNS nutrient databank.

### Definition of eating occasions

The outcome of interest was eating occasion size, measured in kilojoules (kJ). Eating occasions were defined as an occasion in which energy-containing foods or beverages were consumed within the same 15-min period, as defined in previous eating patterns research in children^(11,25–27)^. If two or more items were consumed within 15 min, these were considered a single eating occasion, if > 15 min separated reported items, these were considered separate eating occasions.

### Eating occasion variables

Parents of participants completed a face-to-face computer-assisted personal interview and questionnaires. Parents were asked to record where and with whom (eating companion) each food and beverage was consumed. The original ‘where’ and ‘with whom’ variables were recoded into six and five categories, respectively, similar to previous research^(28)^ (see online supplementary material, Supplemental Table S1 and Table S2). Parents were asked to record whether each food and beverage was eaten sat at the table or watching TV. Where watching TV responses were not specified, we classified these as ‘not watching TV’ (17 % of occasions).

### Individual variables

Individual characteristics such as child’s gender, age (years), ethnicity and total daily EI were available in the NDNS data. Height and weight data were measured by the interviewer and used to derive BMI z-scores using the BMI WHO cut-offs for 2–3-year-olds^(29)^ and UK90 for 4–5-year-olds^(30,31)^. Parental socio-economic status (SES) was indicated by parental occupation using the National Statistics Socio-Economic Classification (NSSEC)^(32)^ (see online supplementary material, Supplemental Table S3). Misreporting of EI was assessed using the individualised method for children^(33,34)^, which involved calculating the ratio of reported EI to estimated energy requirements, accounting for growth. Plausible reporting of EI was identified using cut-offs of 0·79 and 1·21. Seventeen per cent of the total sample were categorised as under-reporters and 20 % as over-reporters.

### Statistical analysis

#### Descriptive analysis

All analyses were conducted in Stata 15. Exposure variables included four eating occasion characteristics (eating contexts): location, eating companion, watching TV, and sitting at a table, and four individual characteristics: age, gender, ethnicity, and parental SES. Descriptive statistics on characteristics of eating occasions were reported at the survey level (across all young children). Number and frequency of eating occasions and median (and interquartile range (IQR)) eating occasion size were reported for each eating context variable. Descriptive statistics on individual characteristics were reported. Number (%) of children was reported for categorical variables and mean and standard deviation for continuous variables. Mean eating occasion frequency and median eating occasion size were reported for categorical variables. For continuous variables, simple regression analyses were conducted, and β-coefficients (B) and 95 % CI were reported. The number of young children who reported to consume an eating occasion in each of the eating contexts was presented across all children and by individual characteristics, to understand how these variables were inter-related.

Energy density of eating occasions (as defined above) was calculated (kilojoules of eating occasion divided by grams of eating occasion) and median (IQR) was reported. Simple analysis of food groups associated with larger eating occasions was conducted. The food groups classified within the NDNS were collapsed further according to the UK Eatwell Guide^(35)^ food groups (starchy, protein, fruit and vegetables, dairy, oils and spreads, foods high in fat and sugar, and drinks)^(19)^. The percentage of all eating occasions in which young children consumed a given food group was reported. Spearman’s correlations were conducted to explore correlations between percentage of total energy consumed in an eating occasion from a food group and overall eating occasion size.

#### Multilevel modelling

Hierarchical multilevel modelling^(36)^ was used to explore the relationship of eating occasion size with eating contexts and individual characteristics as potential exposure variables. Eating occasions (level 1 variation) are nested within children (level 2 variation). Therefore, multilevel modelling allowed us to explore whether eating occasion size varied within and between children, as well as the potential exposures that explained this variability. Eating occasion size (kJ) was not normally distributed and was logged transformed to approximate the normal distribution. Individual-level survey weights from each survey wave were combined according to NDNS instructions^(10)^ and used in analyses to account for selection and non-response biases.

Several models were run: Model 1 was the variance component (null intercept) model, which did not include any exposure variables. This model assessed how much variability in eating occasion size was attributable to within-children-between-eating occasions and between-children variance. Models 1·1 to 1·8 explored the unadjusted associations between each of the eight exposures of interest and eating occasion size in their own model. In Models 2·1 to 2·8, a set of confounders unique to each of the eight exposures of interest were added to each model to explore if the evidence and size of associations were robust to adjustment for potential confounding. Supplemental Table S4 provides a description of each model, including the potential confounders added for each exposure at each stage. Models 2·1 to 2·8 were adjusted for misreporting of EI as a potential confounder, because misreporting has previously been shown to affect diet–health relationships^(37–39)^. Individual-level models (2·5–2·8) were adjusted for total daily EI^(40)^.

For each model, the intraclass correlation and ‘percentage variance explained’ were calculated. These indicated the percentage of variation in eating occasion size attributed to differences at our two levels of variation and how much variance could be explained by our exposure variables compared to the null-intercept model, respectively. Model fit was assessed using likelihood ratio tests. Estimates were converted to kilojoules (kJ) by multiplying the adjusted ratios by the model intercept, to provide meaningful public health units.

The STROBE flowchart^(41)^ (see online supplementary material, Supplemental Fig. S1) illustrates the amount of missing data in the sample. We reported the sample size of each model and used the likelihood ratio test to assess whether missing data could bias our results.

#### Mediation analyses

To aid interpretation of the results from Models 2·1 to 2·8, mediation analysis was conducted. Eating occasion type (whether an eating occasion was defined as a meal or snack) and eating frequency (average number of daily eating occasions) were added as potential mediators to the eating occasion-level and child-level models, respectively, to explore whether potential associations observed were due to children consuming specific eating occasion types or eating more frequently. Each eating occasion was defined as a meal or snack using a time of day, plus energy criterion method based on our data, similar to previous research^(42–44)^. The percentage energy from each eating occasion (of total daily EI) was plotted in 30-min intervals over a 24-h period, across all participants. The resulting graph (see online supplementary material, Supplemental Fig. S2) displayed three peaks in energy across the day, which were used to label eating occasions as meals or snacks. We defined meals as eating occasions with the largest percent energy between 05.30–10.00, 11.00–14.00 and 16.00–19.00. All other smaller eating occasions within these mealtimes and all eating occasions outside of these mealtimes were defined as snacks. In the descriptive results, eating occasion size and frequency were additionally reported for meals and snacks because meals and snacks are systematically different in size. In the multilevel models, potential mediators were added to Models 2·1 to 2·8 if an exposure-outcome association was observed. Estimates from the mediation models were compared to the final adjusted estimates to explore potential mediation. Models 3·1 to 3·8 present the mediation models and are presented as the final models because including eating occasion type and eating frequency provided the most meaningful interpretation of results within the context of the study.

## Results

### Descriptive results

#### Characteristics of eating occasions

The median eating occasion size across all young children (*n* 1962) and all eating occasions (*n* 48 219) was 657 kJ (IQR 356, 1117). The median size for meals was 1050 kJ (IQR 711, 1506) and for snacks was 402 kJ (IQR 209, 640). On average, young children consumed 6·7 (sd 1·8) eating occasions per d, of which 3·0 (sd 0·3) were meals and 3·7 (sd 1·9) were snacks.

Supplemental Table S5 displays the number (%) of eating occasions across the different eating contexts. Nearly three-quarters of eating occasions occurred at home, with 11 % in childcare and just 2 % in eateries. Meals made up 47 % and snacks 53 % of eating occasions overall, whereas 63 % of occasions in eateries were meals and 77 % of eating ‘on the go’ was a snack. Parents and/or other family members ate with young children in 85 % of eating occasions, with just 5 % eaten alone. A third of occasions were while watching TV and nearly half were while sitting at a table. Supplemental Figure S3 illustrates the median (IQR) eating occasion, meal and snack sizes across the different eating contexts.

Supplemental Table S6 suggests larger eating occasions were more energy-dense than smaller eating occasions (4·6 kJ/g *v*. 1·7 kJ/g) and contained more food groups (the percentage of all eating occasions in which young children consumed a given food group was greater across all food groups for larger *v*. smaller eating occasions). Supplemental Table S7 suggests percentage energy from all food groups (but not drinks) were correlated with overall eating occasion size.

#### Characteristics of young children

Table 1 describes the sample of young children (*n* 1962). The sample consisted of 53 % boys, 86 % White ethnicity, 39 % low SES, with a mean child age of 3 years (sd 1·3). Supplemental Figure S4 presents the median (IQR) eating occasion, meal and snack sizes across the individual characteristics. The overall frequency of eating occasions was similar among boys and girls, and SES groups but varied by ethnicity; 7·1 times/d among Asian/Asian British children *v*. 5·6 times/d among Black/Black British children. A lower eating occasion frequency and greater eating occasion size was associated with being older (0·3 eating occasions less per d and 92 kJ more per occasion, per year of age). A higher eating frequency was associated with smaller eating occasions (–67 kJ per occasion for each extra time eating occurred). A higher total EI was associated with larger eating occasions (52 kJ per occasion for each 418 kJ of total energy consumed) (data not shown, table available upon request).

**Table 1:** Characteristics of young children 1–5 years (*n* 1962) in the UK National Diet and Nutrition Survey 2008–2017

	*n*	%	
Gender			
Boys	1034	53	
Girls	928	47	
Socio-economic status			
Low	744	39	
Intermediate	368	19	
High	812	42	
Ethnicity			
White	1688	86	
Black/Black British	49	3	
Asian/Asian British	122	6	
Mixed	64	3	
Other	39	2	
Misreporting of energy intake			
Plausible reporter	1233	63	
Under-reporter	338	17	
Over-reporter	391	20	

	*n*	Mean	sd
Age (years)	1962	3·1	1·3
BMI z-score*	1593	0·4	1·2
Total daily eating frequency	1962	6·7	1·8
Total daily energy intake (kJ)	1962	5012	1163

* Calculated using WHO BMI z-scores for 1·5–3 years and UK 1990 BMI z-score 4–5 years.

#### Characteristics of young children within eating contexts

All young children reported eating at home, 60 % in childcare and 51 % ‘on the go’. Fewer young children ate at a friend’s or relative’s house (46 %), in eateries (34 %) and at activity places (40 %). Eighty per cent of children ate with their parents/carers and 77 % with family and friends. Fewer children ate with parents and siblings (54 %) and with friends (54 %). Only 36 % of children ate alone. Most children reported eating watching TV (92 %) and not watching TV (99 %). Similarly, 96 % of children reported eating sitting at a table and 92 % whilst not. Sitting at a table *v*. not was more common in childcare (68 %) and eateries (74 %); more common between 12.00 and 14.00 (61 %) and less common after 20:00 (17 %); more common when eating with friends (67 %) and less common when eating alone (24 %) (data not shown, table available upon request)

### Multilevel model results

#### Associations of eating contexts with eating occasion size

**Fig. 1:**
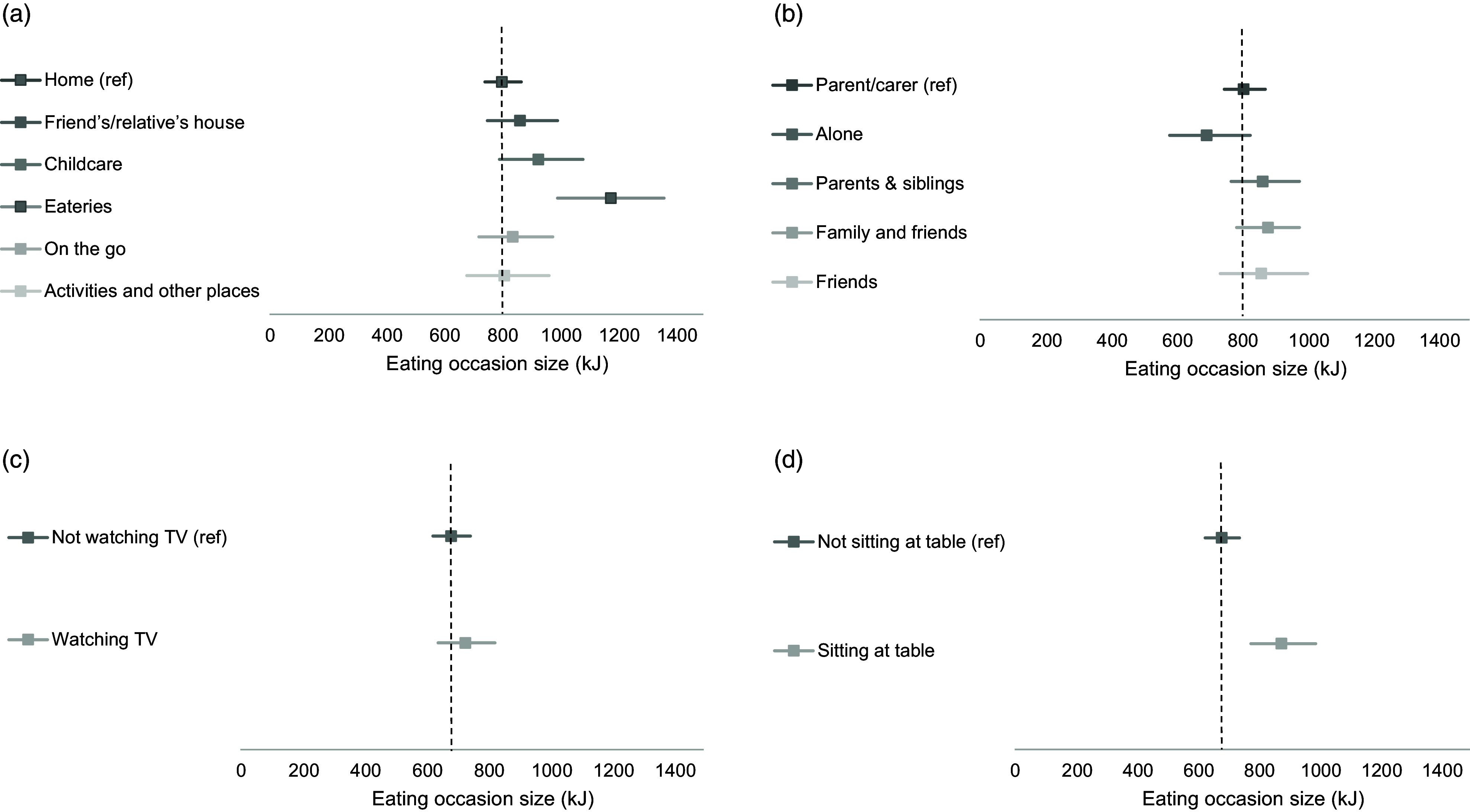
Associations of eating contexts with eating occasion size among young children 1–5 years, in the National Diet and Nutrition Survey 2008–2017. *Computed from Supplemental Table S8 (estimate = intercept × ratio). Estimate shows the eating occasion size (kJ) that young children reported for each eating context. 95 % CI are represented by the horizontal lines. Vertical dashed lines represent the eating occasion size for each reference category (intercept) adjusted for confounders and allows comparison of eating occasion size with other categories

Figure 1 presents the association of eating contexts with eating occasion size in kilojoules from Models 3·1 to 3·4 (adjusted for potential confounders and mediators). Table 2 presents the ratios and 95 % CI from Models 3·1 to 3·4. Model 3 provided the best model fit (see online supplementary material, Supplemental Table S9) and allowed for the most meaningful interpretation of results. Supplemental Table S8 presents the ratios and 95 % CI from Models 1·1 to 1·4 and 2·1 to 2·4 before adjustment for potential mediators. Eating in eateries was associated with the largest eating occasion size in young children, being over 50 % larger than eating at home, equating to a difference of 377 kJ. Eating sitting at a table was associated with a larger eating occasion size; 197 kJ larger *v*. not sitting at a table. Eating in childcare and at a friend’s or relative’s house were associated with larger eating occasion sizes, compared to eating at home (121 kJ and 63 kJ larger, respectively). Eating with parents and siblings, and family and friends were associated with slightly larger eating occasion sizes, equating to 59 kJ and 71 kJ larger than eating with parents only, respectively. Eating alone was associated with smaller eating occasion size; 113 kJ smaller than eating with parents. Eating occasions were slightly larger when watching TV *v*. not, equating to a 46 kJ difference. Eating occasion type was added as a potential mediator to the models to account for meals potentially being more frequently consumed in certain eating contexts than snacks and therefore explaining why eating occasion size is larger (because meals are systematically larger than snacks). After adding eating occasion type, eating on-the-go, at activity places and with friends were no longer associated with eating occasion size (fully mediated relationship). Estimates were partially mediated after adding eating occasion type for the sitting at a table and eating companion variables (Table 2, see online supplementary material, Supplemental Table S8).

**Table 2:** Relationship of eating occasion size with eating contexts and individual characteristics among young children 1–5 years (*n* 1962) in the UK National Diet and Nutrition Survey 2008–2019. Presents results from Model 3

	Models 3·1–3·8 – Adjusted for potential confounders and mediators (eating occasion type and eating frequency)		
Exposure	Ratio*	95 % CI	*P*-value
Eating contexts			
Model 3·1 – location†			
Home (ref)	1·00		
Friend’s/relative’s house	1·08	1·02, 1·14	0·014
Childcare	1·15	1·07, 1·24	< 0·001
Eateries	1·47	1·34, 1·56	< 0·001
On the go	1·05	0·98, 1·13	0·199
Activities and other places	1·01	0·92, 1·11	0·857
Model 3·2 – eating companion‡			
Parent/carer (ref)	1·00		
Alone	0·86	0·78, 0·95	0·002
Parents and siblings	1·07	1·03, 1·12	0·002
Family and friends	1·09	1·05, 1·13	< 0·001
Friends	1·06	0·99, 1·15	0·114
Model 3·3 – watching TV whilst eating§			
Not Watching TV (ref)	1·00		
Watching TV	1·07	1·03, 1·11	0·001
Model 3·4 – sitting at table whilst eating||			
Not sitting at table (ref)	1·00		
Sitting at table	1·29	1·24, 1·34	< 0·001
Individual characteristics			
Model 3·5 – age¶			
1 year (ref)	1·00		
2 years	1·04	0·94, 1·14	0·479
3 years	0·97	0·87, 1·07	0·541
4 years	1·00	0·89, 1·13	0·947
5 years	1·00	0·89, 1·12	0·995
Model 3·6 – gender¶			
Boys (ref)	1·00		
Girls	0·99	0·95, 1·02	0·407
Model 3·7 – ethnicity¶			
White (ref)	1·00		
Black/Black British	1·20	1·12, 1·27	< 0·001
Asian/Asian British	1·19	1·12, 1·26	< 0·001
Mixed	1·16	1·09, 1·23	< 0·001
Other	1·23	1·14, 1·32	< 0·001
Model 3·8 – parental SES**			
Low (ref)	1·00		
Intermediate SES	1·02	0·96, 1·08	0·518
High SES	1·04	0·99, 1·08	0·118

SES, socio-economic status.

* To improve interpretability, ratios are presented as the exponentiated values of the log-transformed coefficients and represent changes in the ratio of the mean eating occasion size. For example, an exponentiated value of 1·14 represents a 14 % difference in eating occasion size between the specified eating context/individual characteristic and its reference category.

† Adjusted for time of day, day of week, day number, age, ethnicity, parental SES and misreporting (and eating occasion type as potential mediator).

‡ Adjusted for time of day, day of week, location, day number, age, ethnicity and misreporting (and eating occasion type as potential mediator).

§ Adjusted for time of day, day of week, location, eating companion, sitting at the table, day number, age, ethnicity, parental SES and misreporting (and eating occasion type as potential mediator).

|| Adjusted for time of day, day of week, location, eating companion, watching TV, day number, age, ethnicity and misreporting (and eating occasion type as potential mediator).

¶ Adjusted for misreporting, total daily energy intake and zBMI (and eating frequency as potential mediator).

** Adjusted for misreporting, total daily energy intake, ethnicity and zBMI (and eating frequency as potential mediator).

#### Associations of individual characteristics with eating occasion size

**Fig. 2:**
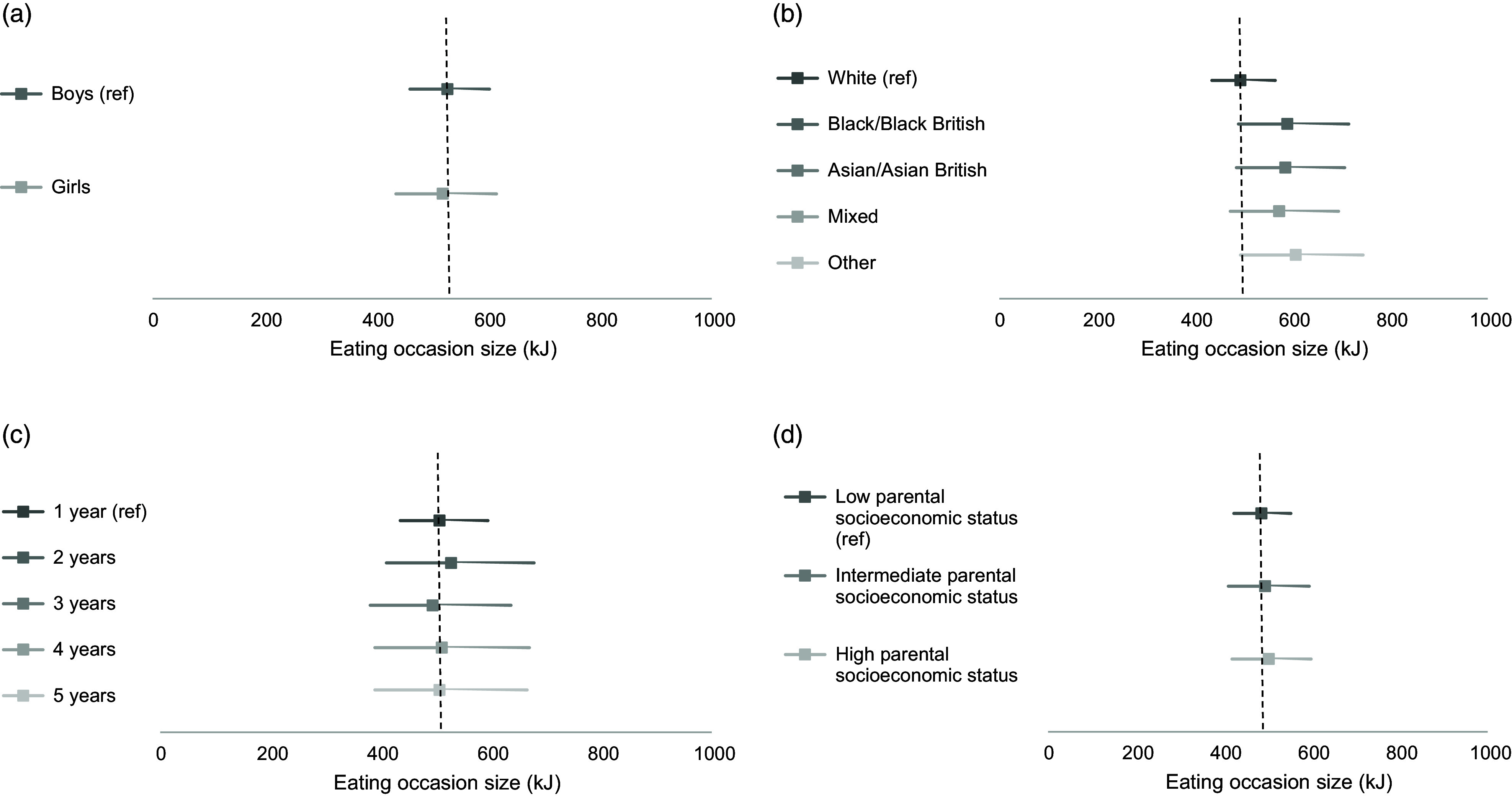
Associations of individual characteristics with eating occasion size among young children 1–5 years, in the National Diet and Nutrition Survey 2008–2017. *Computed from Table S8 (estimate = intercept × ratio). Estimate shows the eating occasion size (kJ) that young children report for each individual characteristic. 95 % CI are represented by the horizontal lines. Vertical dashed lines represent the eating occasion size for each reference category (intercept) adjusted for confounders and allows comparison of eating occasion size with other categories

Figure 2 presents the association of individual characteristics with eating occasion size in kilojoules from Models 3·5 to 3·8. Table 2 presents the ratios and 95 % CI from Models 3·5 to 3·8. Young children of Black, Asian, Mixed and Other ethnicities had eating occasion sizes slightly larger than children of White ethnicity, by 96 kJ, 92 kJ, 79 kJ and 113 kJ, respectively. Gender and parental SES showed no evidence of association with eating occasion size. Eating frequency was added as a potential mediator to the models to account for eating occasion size being larger due to eating less frequently. After adding eating frequency, being older was no longer associated with eating occasion size (fully mediated relationship) (Table 2, see online supplementary material, Supplemental Table S8).

#### Explaining eating occasion size variation by eating occasion *v*. child characteristics

Supplemental Table S9 presents the variance estimates for each of the multilevel models. The null-intercept model showed most of the variation in eating occasion size was attributed to characteristics of the eating occasion (90 % variance), leaving just 10 % variation attributable to characteristics of the young children. Eating contexts (location, eating companion, watching TV and sitting at a table) explained 16 % of the total variance in eating occasion size, whereas the individual characteristics (age, gender, ethnicity and parental SES) explained just 2 %. When all exposures and confounders were added, total variance explained was 23 %, which increased to 41 % when mediators (eating occasion type and eating frequency) were added.

## Discussion

Ninety per cent of the variation in eating occasion size was within children, with only 10 % attributed to differences between children. Eating contexts explained 16 % of the total variance in eating occasion size, compared to only 2 % explained by individual characteristics. These findings suggest factors that differ from one occasion to another (such as eating contexts) can better help us to understand why portion sizes are larger in this sample of young children than factors that differ from one child to another (such as individual characteristics). Our findings align with Toumpakari et al.^(13)^ who found 89 % of variation in non-core EI in adolescents was attributed to characteristics of the eating occasions. We therefore support future research and guidance to focus on the eating environment in young children^(19,45)^.

Public Health England (PHE) recommended children should only consume two 100 kcal (418 kJ) snacks (excluding fruit and vegetables) per d^(46)^. Our findings suggest young children in this sample, on average consumed more than three 402 kJ snacks per d, which could exceed PHE recommendations. Median meal size was 1050 kJ. A systematic review of resources recommending portion sizes for 1–5-year-olds found recommended meal sizes across resources were between 473 kJ and 1761 kJ^(19)^. A comparison should be interpreted with caution because several resources included in the review recommended portion sizes to meet energy requirements of 3–5-year-olds (whereas this sample also included younger children, with lower energy requirements). If following certain recommendations, such as those from the Infant and Toddler Forum^(47)^, young children in this sample could be consuming larger meals than recommended. This highlights the need to promote consumption of age-appropriate portion sizes to meet energy requirements.

Sitting at a table was independently associated with an eating occasion size on average 197 kJ larger than not sitting at a table. Compared to eating with parents only, eating occasion size was larger when eating with parents and siblings and with family and friends, by 59 kJ and 71 kJ, respectively. Compared to eating at home, eating occasion size was larger when eating in childcare and at a friend’s or relative’s house, by 121 kJ and 63 kJ, respectively. Although portion sizes may be larger in these contexts, this is only problematic if portions are large enough to result in surplus EI, as this could lead to excessive weight gain^(17)^. It may be larger portions of healthy foods, such as vegetables are being consumed in these contexts^(14)^, due to larger servings^(48)^, or consumption being encouraged and modelled by others^(49)^. However, large portions of vegetables are not likely to result in energy-dense eating occasions because of their low-energy content. If high energy-dense foods such as desserts are being consumed in these contexts^(50)^, this could substantially increase the energy content of an eating occasion and lead to a surplus EI. Our results suggest larger eating occasions were more energy-dense, contained more food groups and were being driven the most by a greater percentage energy from starchy foods and proteins (see online supplementary material, Supplemental Table S6 and S7). The findings suggest parents and childcare settings may need education on how to achieve balanced meals containing appropriately sized portions across food groups.

We accounted for other eating contexts and individual characteristics that could have been associated with eating at a table, with others and out of home (such as sitting at a table being more likely in eateries and during lunch, when meals are larger)^(51,52)^. Parental feeding styles and practices^(7)^, modelling behaviours^(53)^ and how much parents serve themselves^(54)^ influence what and how much young children eat. Certain practices and behaviours, such as encouraging plate cleaning, can lead parents to override their children’s ability to self-regulate their intake, leading to long-term over-consumption^(53)^. It may be that when children eat at the table with their parents (and others), the social influences contribute towards consuming more. Interventions targeting parent feeding styles^(55,56)^ should incorporate portion size advice to help promote children’s self-regulation from a young age.

Eating in eateries (such as cafes, fast-food outlets and restaurants) was independently associated with the highest eating occasion size; on average, 377 kJ larger than eating at home. This is not surprising considering the existing literature suggesting restaurant meals (including children’s meals) are large in portion size, too energy-dense^(51)^ and do not meet nutritional standards^(57)^. In addition, eateries are associated with higher consumption of ultra-processed^(58)^ and non-core^(13)^ food, in children and adolescents. In our sample, only 34 % of young children ate at eateries (only 2 % of the total number of eating occasions). Similarly, Mak et al.^(14)^ found only 2·3 % of the total eating occasions were consumed in eateries among 7–10-year-olds. However, large portion sizes served in eateries could influence consumption norms, by distorting both parents’ and children’s understanding of appropriate portion sizes^(59)^, especially when children are more susceptible to consuming large portions^(7)^. As stated in the UK Childhood Obesity plan^(60)^, reducing energy content (and therefore portion size) of meals served in eateries could be a target for action. The number of out-of-home eating occasions may have been under-reported by parents due to the increased burden^(61)^. However, given the small number of eating occasions that were reported in eateries, to have a greater effect on reducing child population-level portion sizes, it may be more appropriate to target the home and childcare environments, where we found more meals and snacks were consumed (73 % and 11 %, respectively).

Although we and others^(11,62)^ have identified individual characteristics and eating contexts associated with consuming larger portions, our models only explained 41 % of the total variation. This suggests there are several other factors that need to be identified to fully explain why portion size varies in young children. A child’s susceptibility to consume large portion sizes is due to a complex combination of nature (e.g. genetics), nurture (e.g. parent feeding practices), individual traits (e.g. satiety) and the environment (e.g. home food environment)^(7)^. Child-related factors such as eating traits and liking of the food, caregiver-related factors such as caregiver portion sizes and feeding practices^(63)^, and food-related factors such as energy density^(64)^ may all interact to influence the portion sizes children consume. Our results suggest we should focus on building the evidence base for factors associated with portion size that vary from one eating occasion to another (because this is where most of the variation in eating occasion size lies). Factors such as the food environment, child temperament, hunger and liking, parental feeding practices, serving method and food type should be further explored, whilst also considering how individual traits and characteristics may be bidirectionally related^(7)^.

Experimental portion size manipulation studies have shown significant increases in EI from a meal or snack, as a result of serving large portion sizes, by between 63 and 347 kJ^(4,5,64–69)^. Although we do not have data on the served portions and the data on consumed portions is an estimate of EI, we observed associations, which equated to eating occasions being between 46 and 377 kJ larger than reference category eating occasions. The difference in kilojoules is relatively small when comparing one eating context or child to another. However, if young children consistently consume meals or snacks in certain contexts or because of individual characteristics, which are associated with larger portions, this may have implications for excessive EI over time, and excessive weight gain^(17,70)^. Hebestreit et al.^(71)^ found daily food intake (g) and total EI (kcal) were positively associated with BMI z-score in 2–9-year-old children. However, in a combined model, only total EI was independently associated. As portion size is highly related to EI, the consistent consumption of large energy-dense portion sizes may contribute to excessive total EI and weight gain over time^(17)^. Therefore, the focus on age-appropriate portion sizes and EI is critical^(72)^. In addition, caregivers may benefit from guidance highlighting how the food environment can encourage the consumption of larger portions.

### Strengths and limitations

Using multilevel modelling, we have accounted for the clustered hierarchical nature of our data (whereby eating occasions are nested within individuals). The multilevel models have accounted for both within and between variation in eating occasion size in young children, which minimises the potential biases related to person-level unmeasured variables associated with our outcome variable^(58)^. We used combined data from the NDNS Years 1–9, which enabled analysis of a large, UK nationally representative sample. We considered each exposure variable as a separate model to ensure appropriate adjustment for confounders and mediators, and to increase reliability.

The main limitation was energy density of and types of foods in the eating occasions were not analysed in detail, which limits our interpretations. Although based on previous research^(42,73)^ and preliminary work, our chosen definition of eating occasion type may have inaccurately classified some eating occasions as meals and snacks and affected estimates. Despite this, Model 3, which included eating occasion type and eating frequency as potential mediators, was presented as the final model. Model 2 was provided for comparison between models. Including eating occasion type in Model 3 provided a more meaningful interpretation of the estimates, because only meals were compared to meals and only snacks compared to snacks, which accounted for the systematic difference in size between meals and snacks. For example, eating on-the-go was associated with a smaller eating occasion size in Model 2 but in Model 3, when accounting for snacks being the predominant eating occasion on-the-go, an association was no longer observed. Similarly, including eating frequency in Model 3 provided more meaningful interpretation, because the size of an eating occasion may depend on how frequently a child eats.

Due to the cross-sectional nature of the data, our findings do not provide evidence of causation. Our sample included mostly White British young children (86 %) and so the findings may be less generalisable to other ethnic groups. Although, misreporting of EI was calculated and added to models, the parent-reported dietary data were subject to misreporting and subject bias^(74)^. The variables we selected for analysis only explained 41 % of the variation in eating occasion size, which limits our interpretations. The survey lacked data on appetite traits and parental feeding behaviours^(7)^, which may have improved the percentage variance explained and enhanced our interpretations.

### Future research and policy implications

Future research should continue to focus on eating habits of children and how these may affect EI, dietary intake and weight gain. Future research should pull together data or create new datasets that include all the factors previously associated with portion size in children, to better understand which factors have the greatest influence on increasing children’s susceptibility to consuming larger portions. Future research should explore how the portion sizes of specific food groups or individual foods are combined and how they contribute to large eating occasions. It is also important to establish an accepted consensus for classifying eating occasions as meals and snacks where participant-reported eating occasions are not available. Future research should compare consumed meal and snack sizes reported in national surveys with the recommendations, to establish whether young children are overconsuming.

Governments and food industries should work together to agree on policies to reduce out-of-home portion sizes of children’s meals and snacks. This could be achieved through the combination of reducing dishware and packet sizes^(75,76)^, introducing calorie caps on meals in eateries (similar to the UK Soft Drinks Industry Levy)^(77)^ and/or price incentives for selecting smaller portions^(78)^.

## Conclusion

To conclude, the variability in eating occasion size in young children is better explained by differences between eating occasions rather than individuals. Efforts to reduce portion sizes in children should focus on eating contexts rather than targeting children with certain demographic characteristics. Eating in eateries, sitting at a table, in childcare, with other family members and friends, and watching TV were all eating contexts associated with larger eating occasions. Effective strategies to promote the consumption of age-appropriate portion sizes, especially in the home environment, should be developed.

## Supplementary material

For supplementary material accompanying this paper visit https://doi.org/10.1017/S1368980021005024. click here to view supplementary material
