# Supplementary material for: Where and when are portion sizes larger in young children? An analysis of eating occasion size among 1·5–5-year-olds in the UK National Diet and Nutrition Survey (2008–2017)
Source: Public Health Nutr. 2021 Dec 27;25(12):3420–31. doi: 10.1017/S1368980021005024 (PMC9991682; doi:10.1017/S1368980021005024)
Supplement: Supplementary file 1 [file S1368980021005024sup001.docx]

# Supplementary material

## Supplementary methods

### Table S1. Breakdown of original Location (‘Where’) eating context

| **Home** | Home - Bedroom |
| --- | --- |
|  | Home - Dining Room |
|  | Home - Garden |
|  | Home - Kitchen |
|  | Home - Living Room |
|  | Home - Other |
|  | Home - Unspecified |
| **Friend's or Relative's house** | Holiday Accommodation |
|  | Friend's or Relative's house |
| **Childcare** | School - Canteen - Bought food |
|  | School - Canteen - Food from home |
|  | School - Canteen - Other |
|  | School - Classroom |
|  | School - Other |
|  | School – Playground |
|  | Carer's home |
|  | Nursery/Kindergarten |
| **Eateries** | Coffee shop, cafe, shop, deli, sandwich |
|  | Fast food outlet |
|  | Restaurant, pub, night club |
| **On the go** | Not At Home - Unspecified |
|  | Outside - Other |
|  | Street |
|  | Bus, car, train |
| **Activity and other places** | Leisure Activities, shopping, tourist a |
|  | Sports club, sports leisure venue |
|  | Community Centre/Day Centre/Drop in |
|  | Community centre/day centre/drop-in |
|  | Place of Worship |
|  | Public Hall/Function Room |
|  | Other place |
|  | Unspecified |
|  | Work - Canteen - Bought food |
|  | Work - Canteen - Food from home |
|  | Work - Canteen - Other |
|  | Work - Desk |
|  | Work - Other |

### Table S2. Breakdown of original Eating companion (‘with whom’) eating context

| **Alone** | Alone |
| --- | --- |
| **Parents/carers** | Parent(s)/Carer |
|  | Partner |
| **Parents & siblings** | Parent(s)/Carer & Siblings |
|  | Siblings |
| **Family & friends** | Family (incl. Relatives) |
|  | Family & Friends |
|  | Carer & Other Children |
|  | Partner & Children |
| **Friends** | Friends |
|  | Child/Children |
|  | Others - General Public |
|  | Others General public |
|  | Others - Known to Respondent |
|  | Others known to Respondent |
|  | Work colleagues |
| **Not specified** | Not specified |

*’Not specified’ recoded as missing (13% of occasions)

### Table S3. Breakdown of Parental SES categories using the National Statistics Socio-economic classifications (NS-SEC)

| **High** | Higher managerial, administrative and professional occupations |
| --- | --- |
|  | Large employers and higher managerial and administrative occupations |
|  | Higher professional occupations |
|  | Lower managerial, administrative and professional occupations |
| **Intermediate** | Intermediate occupations |
|  | Small employers and own account workers |
| **Low** | Lower supervisory and technical occupations |
|  | Semi-routine occupations |
|  | Routine occupations |
|  | Never worked and long-term unemployed |

### Table S4. Description of all multilevel models in analyses of variation in eating occasion size in young children

| **Model** | **Description** | |
| --- | --- | --- |
| Model 1 | Random intercept null model. Includes no explanatory variables. Purpose is to partition within and between person variation using eating occasion size as the level one (within) variable and participant ID as the level two (between) variable | |
| **Unadjusted models (1.1-1.8)** to estimate the unadjusted associations between eating occasion size and potential exposure variables | | |
| Model 1.1 | Location of eating occasion (5 dummy variables) | |
| Model 1.2 | Eating companion at eating occasion (5 dummy variables) | |
| Model 1.3 | Watching TV eating at an eating occasion | |
| Model 1.4 | Sitting at table eating at an eating occasion | |
| Model 1.5 | Age (5 dummy variables) | |
| Model 1.6 | Gender | |
| Model 1.7 | Ethnicity (5 dummy variables) | |
| Model 1.8 | Parental socioeconomic status (3 dummy variables) | |
| **Adjusted models (2.1-2.8)** to estimate the adjusted associations of the main exposure variables independent of potential confounding | | |
|  | **Main exposure** | **Covariates** |
| Model 2.1 | Location | time of day, day of week, day number, age, ethnicity, parental SES, and misreporting |
| Model 2.2 | Eating companion | time of day, day of week, location, day number, age, ethnicity, and misreporting |
| Model 2.3 | Watching TV eating | time of day, day of week, location, eating companion, sitting at the table, day number, age, ethnicity, parental socioeconomic status, and misreporting |
| Model 2.4 | Sitting at a table eating | time of day, day of week, location, eating companion, watching TV, day number, age, ethnicity, and misreporting |
| Model 2.5 | Age | misreporting, total daily energy intake, and zBMI |
| Model 2.6 | Gender | misreporting, total daily energy, intake and zBMI |
| Model 2.7 | Ethnicity | misreporting, total daily energy intake, and zBMI |
| Model 2.8 | Parental socioeconomic status | ethnicity, misreporting, total daily energy intake, and zBMI |
| **Mediation models (3.1-3.8)** to estimate the mediating effect on the associations between exposure variables and eating occasion size, after adjusting for potential confounding | | |
|  | **Main exposure** | **Potential mediator** |
| Model 3.1 | Location | Eating occasion type |
| Model 3.2 | Eating companion | Eating occasion type |
| Model 3.3 | Watching TV eating | Eating occasion type |
| Model 3.4 | Sitting at a table eating | Eating occasion type |
| Model 3.5 | Age | Eating frequency |
| Model 3.6 | Gender | Eating frequency |
| Model 3.7 | Ethnicity | Eating frequency |
| Model 3.8 | Parental socioeconomic status | Eating frequency |

### Figure S1. STROBE flowchart of missing data, illustrating reduction in sample size as potential correlates are added to the models

N eating occasions for entire survey sample = 48,419

N (%) eating occasions for sub-sample with complete data for all eating occasions-level correlates = 42,219 (87%)

N (%) eating occasions for sub-sample with complete data for all correlates (eating occasion-level and person-level) = 33,565 (69%)

### Figure S2. Percentage energy of eating occasions consumed by time of day to classify eating occasions as meals and snacks

## Supplementary results

### Table S5. Characteristics of eating occasions in absolute frequencies and percentages and median eating occasion size of eating contexts, among young children 1-5 years (n 1962), in the UK National Diet and Nutrition Survey 2008-17

|  | **Total eating occasions N (%)** | | |
| --- | --- | --- | --- |
|  | **Total** | **Meal** | **Snack** |
| Overall | 48419 (100) | 22836 (47) | 25583 (53) |
| Location |  |  |  |
| Home | 35294 (73) | 17290 (76) | 18004 (70) |
| Friend’s/relative’s house | 3070 (6) | 1450 (6) | 1620 (6) |
| Childcare | 5333 (11) | 2451 (11) | 2882 (11) |
| Eateries | 974 (2) | 620 (3) | 354 (1) |
| On the go | 2191 (5) | 504 (2) | 1687 (7) |
| Activities and other places | 1557 (3) | 521 (2) | 1036 (4) |
| Eating companion |  |  |  |
| Parents/carers | 15337 (36) | 7267 (35) | 8070 (38) |
| Alone | 2202 (5) | 673 (3) | 1529 (7) |
| Parents & siblings | 10065 (24) | 5627 (27) | 4438 (21) |
| Family and friends | 10250 (25) | 5307 (25) | 4943 (23) |
| Friends | 4365 (10) | 2002 (10) | 2363 (11) |
| Watching TV |  |  |  |
| No | 31960 (66) | 15301 (67) | 16659 (65) |
| Yes | 16459 (34) | 7535 (33) | 8924 (35) |
| Sitting at table |  |  |  |
| No | 16432 (34) | 4564 (20) | 11868 (46) |
| Not Specified | 9209 (19) | 3112 (14) | 6097 (24) |
| Yes | 22778 (47) | 15160 (66) | 7618 (30) |
| Time of day |  |  |  |
| 06:00-09:00 | 7868 (16) | 5595 (25) | 2273 (9) |
| 09:00-12:00 | 8940 (18) | 2960 (13) | 5980 (23) |
| 12:00-14:00 | 8127 (17) | 6267 (27) | 1860 (7) |
| 14:00-17:00 | 8920 (18) | 1577 (7) | 7343 (29) |
| 17:00-20:00 | 11289 (23) | 6429 (28) | 4860 (19) |
| 20:00-22:00 | 2789 (6) | 0 (0) | 2789 (11) |
| 22:00-06:00 | 486 (1) | 8 (0.04) | 478 (2) |
| Day of the week |  |  |  |
| Weekday | 33550 (69) | 15850 (69) | 17700 (69) |
| Weekend | 14869 (31) | 6986 (31) | 7883 (31) |

### Figure S3. Median (IQR) size of eating occasions and eating occasions defined as meals and snacks across eating contexts (location, eating companion, watching TV and sitting at a table) among young children 1-5 years (n 1962), in the UK National Diet and Nutrition Survey 2008-17

Figure S3A. Median (IQR) eating occasion, meal, and snack sizes for locations that young children eat at

Figure S3B. Median (IQR) eating occasion, meal, and snack sizes for who young children eat with (eating companions)

Figure S3C. Median (IQR) eating occasion, meal, and snack sizes for when young children watch TV versus do not watch TV whilst eating

Figure S3D. Median (IQR) eating occasion, meal, and snack sizes for when young children sit at a table versus do not sit at a table whilst eating

### Figure S4. Median (IQR) size of eating occasions and eating occasions defined as meals and snacks among young children 1-5 years (n 1962) by individual characteristics (gender, ethnicity, age and parental socioeconomic status), in the UK National Diet and Nutrition Survey 2008-17

Figure S4A. Median (IQR) eating occasion, meal, and snack sizes for boys versus girls

Figure S4B. Median (IQR) eating occasion, meal, and snack sizes by ethnicity

Figure S4C. Median (IQR) eating occasion, meal, and snack sizes by age

Figure S4D. Median (IQR) eating occasion, meal, and snack sizes by parental socioeconomic status

### Table S6. Percentage of all eating occasions in which young children consumed a given food group and the median energy density of an eating occasion for an average, small, and large sized eating occasions

| **Percentage of all eating occasions in which young children consumed a given food group, for all, small, and large sized eating occasion** | | | |
| --- | --- | --- | --- |
|  | All eating occasions | Small eating occasions (lower tertile) | Large eating occasions (upper tertile) |
| **Food group within eating occasions** | % of all eating occasions in which young children consumed a given food group | % of all eating occasions in which young children consumed a given food group | % of all eating occasions in which young children consumed a given food group |
| Dairy | 15 | 6 | 26 |
| Starchy | 45 | 10 | 80 |
| Fruit & Veg | 35 | 29 | 47 |
| Proteins | 23 | 3 | 51 |
| Oils & Spreads | 18 | 3 | 35 |
| High fat, high sugar | 31 | 19 | 41 |
| Drinks | 54 | 41 | 61 |
| **Median energy density of an eating occasion, for an average, small, and large sized eating occasions** | | | |
| **Energy density (kJ/g)** | Median (IQR) | Median (IQR) | Median (IQR) |
| Energy density of eating occasions | 2.1 (1.6, 5.4) | 1.7 (0.4, 3.3) | 4.6 (3.3, 6.3) |

### Table S7. Spearman's correlations between eating occasion size and the percentage energy from food groups within an eating occasion

|  | **Eating occasion size** | |
| --- | --- | --- |
|  | Spearman's correlation | p value |
| **Percentage energy from food groups** | | |
| Dairy (% energy) | 0.2373 | p<0.001 |
| Starchy (% energy) | 0.5063 | p<0.001 |
| Fruit & Veg (% energy) | 0.0872 | p<0.001 |
| Proteins (% energy) | 0.4736 | p<0.001 |
| Oils & Spreads (% energy) | 0.3417 | p<0.001 |
| High fat, high sugar (% energy) | 0.1535 | p<0.001 |
| Drinks (% energy) | -0.0013 | 0.7749 |

### Table S8. Relationship of eating occasion size with eating contexts and individual characteristics across among young children 1-5 years (n 1962), in the UK National Diet and Nutrition Survey 2008-17. Presents results from Models 1 and 2 before adjustment for potential mediators.

*To improve interpretability, ratios are presented as the exponentiated values of the log-transformed coefficients and represent changes in the ratio of the mean eating occasion size. For example, an exponentiated value of 1·14 represents a 14% difference in eating occasion size between the specified eating context/individual characteristic and its reference category.

†adjusted for time of day, day of week, day number, age, ethnicity, parental SES and misreporting, ‡adjusted for time of day, day of week, location, day number, age, ethnicity and misreporting, §adjusted for time of day, day of week, location, eating companion, sitting at the table, day number, age, ethnicity, parental SES and misreporting, |adjusted for time of day, day of week, location, eating companion, watching TV, day number, age, ethnicity and misreporting, ¶adjusted for misreporting, total daily energy intake and zBMI, **adjusted for misreporting, total daily energy intake, ethnicity and zBMI

|  | **Models 1.1-1.8 - Unadjusted** | | | | **Models 2.1-2.8- Adjusted for potential confounders** | | | |
| --- | --- | --- | --- | --- | --- | --- | --- | --- |
| **Exposure** | **Ratio*** | **95% confidence intervals** | | **p value** | **Ratio*** | **95% confidence intervals** | | **p value** |
| **Eating contexts** |  |  |  |  |  |  |  |  |
| **Model .1** - Location† |  |  |  |  |  |  |  |  |
| Home (ref) | 1.00 |  |  |  | 1.00 |  |  |  |
| Friend’s/relative’s house | 1.11 | 1.04 | 1.18 | 0.001 | 1.04 | 0.97 | 1.11 | 0.257 |
| Childcare | 1.07 | 1.02 | 1.13 | 0.009 | 1.09 | 1.00 | 1.18 | 0.043 |
| Eateries | 1.88 | 1.72 | 2.05 | <0.001 | 1.62 | 1.48 | 1.77 | <0.001 |
| On the go | 0.77 | 0.71 | 0.82 | <0.001 | 0.84 | 0.78 | 0.91 | <0.001 |
| Activities and other places | 0.74 | 0.67 | 0.83 | <0.001 | 0.88 | 0.79 | 0.98 | 0.025 |
| **Model .2** - Eating companion‡ |  |  |  |  |  |  |  |  |
| Parent/carer (ref) | 1.00 |  |  |  | 1.00 |  |  |  |
| Alone | 0.71 | 0.63 | 0.79 | <0.001 | 0.74 | 0.66 | 0.82 | <0.001 |
| Parents & siblings | 1.27 | 1.20 | 1.33 | <0.001 | 1.18 | 1.13 | 1.24 | <0.001 |
| Family and friends | 1.28 | 1.23 | 1.34 | <0.001 | 1.19 | 1.14 | 1.25 | <0.001 |
| Friends | 1.17 | 1.10 | 1.25 | <0.001 | 1.12 | 1.02 | 1.22 | 0.014 |
| **Model .3 -** Watching TV whilst eating§ |  |  |  |  |  |  |  |  |
| Not Watching TV (ref) | 1.00 |  |  |  | 1.00 |  |  |  |
| Watching TV | 0.95 | 0.91 | 0.99 | 0.008 | 1.07 | 1.03 | 1.12 | 0.001 |
| **Model .4 -** Sitting at table whilst eating\| |  |  |  |  |  |  |  |  |
| Not sitting at table (ref) | 1.00 |  |  |  | 1.00 |  |  |  |
| Sitting at table | 2.08 | 2.00 | 2.17 | <0.001 | 1.79 | 1.72 | 1.88 | <0.001 |
| **Individual characteristics** |  |  |  |  |  |  |  |  |
| **Model .5 -** Age¶ |  |  |  |  |  |  |  |  |
| 1 year (ref) | 1.00 |  |  |  |  |  |  |  |
| 2 years | 1.20 | 1.08 | 1.32 | <0.001 | 1.24 | 1.08 | 1.43 | 0.002 |
| 3 years | 1.18 | 1.07 | 1.31 | 0.002 | 1.18 | 1.03 | 1.35 | 0.020 |
| 4 years | 1.48 | 1.34 | 1.64 | <0.001 | 1.36 | 1.17 | 1.57 | <0.001 |
| 5 years | 1.63 | 1.47 | 1.79 | <0.001 | 1.44 | 1.24 | 1.66 | <0.001 |
| **Model .6 -** Gender¶ |  |  |  |  |  |  |  |  |
| Boys (ref) | 1.00 |  |  |  | 1.00 |  |  |  |
| Girls | 0.95 | 0.90 | 1.01 | 0.094 | 1.00 | 0.95 | 1.05 | 0.896 |
| **Model .7 -** Ethnicity¶ |  |  |  |  |  |  |  |  |
| White (ref) | 1.00 |  |  |  | 1.00 |  |  |  |
| Black/Black British | 1.38 | 1.22 | 1.55 | <0.001 | 1.37 | 1.24 | 1.52 | <0.001 |
| Asian/Asian British | 1.07 | 0.97 | 1.17 | 0.172 | 1.10 | 1.00 | 1.21 | 0.043 |
| Mixed | 1.21 | 1.05 | 1.40 | 0.010 | 1.24 | 1.10 | 1.39 | <0.001 |
| Other | 1.09 | 0.97 | 1.22 | 0.142 | 1.20 | 1.06 | 1.36 | 0.004 |
| **Model .8 -** Parental SES** |  |  |  |  |  |  |  |  |
| Low (ref) | 1.00 |  |  |  | 1.00 |  |  |  |
| Intermediate SES | 0.98 | 0.92 | 1.05 | 0.584 | 1.00 | 0.94 | 1.07 | 0.977 |
| High SES | 1.00 | 0.95 | 1.05 | 0.965 | 1.00 | 0.95 | 1.06 | 0.980 |

### Table S9. Within and between-person and total variance explained across the different models among young children 1-5 years (n 1962), in the UK National Diet and Nutrition Survey 2008-17

|  | **Null intercept** | **Models 1.1-1.8 - Unadjusted** | | **Models 2.1-2.8 – Adjusted for potential confounders** | **Models 3.1-3.8 – Adjusted for potential confounders and mediators** |
| --- | --- | --- | --- | --- | --- |
|  |  | **Eating contexts** | **Individual characteristics** | **All exposures and confounders** | **All exposures, confounders, and mediators** |
| **Variance (%)*** |  |  |  |  |  |
| Between-child variance explained (%)† | n/a | 22 | 19 | 55 | 84 |
| Within-child-between EO variance explained (%)† | n/a | 16 | 0 | 20 | 36 |
| Total variance explained (%)† | n/a | 16 | 2 | 23 | 41 |
| Log maximum likelihood | 155890 | 127359** | 153548** | 99677** | 91736** |
| N young children | 1,962 | 1,955 | 1,924 | 1,558 | 1,558 |
| N eating occasions | 48,419 | 42,225 | 47,559 | 33,578 | 33,578 |

*Inter-class correlation (ICC) calculated and multiplied by 100 to give percentage variance

†Calculated as percentage change from Model 1 (null intercept model with no explanatory variables)

**P<0.001. A likelihood ratio test was conducted by comparing the deviance across models
